# Cost-effectiveness of telehealth in people with social care needs: the Whole Systems Demonstrator cluster randomised trial

**Published:** 2012-06-15

**Authors:** Catherine Henderson, Martin Knapp, José-Luis Fernández, Jennifer Beecham, Shashivadan Hirani, Michelle Beynon, Martin Cartwright, Lorna Rixon, Stanton Newman, Helen Doll

**Affiliations:** London School of Economics, UK; London School of Economics, UK; London School of Economics, UK; London School of Economics, UK; Health Services Research, City University, London, UK; Health Services Research, City University, London, UK; Health Services Research, City University, London, UK; Health Services Research, City University, London, UK; Health Services Research, City University, London, UK; Department of Public Health, University of Oxford, UK

**Keywords:** economic evaluation, social care, cost analysis, telecare

## Abstract

**Introduction:**

The Whole Systems Demonstrator pilots introduced telehealth and telecare into three local authority areas using an integrated approach to deliver health and social care to those with high care needs and long-term conditions. Proponents of these technologies have given cost savings as one rationale for advocating their introduction and widespread implementation; proponents have also advocated their potential to improve the quality of life for their users. Until recently, evaluations of telehealth and telecare in high-income countries have been based on relatively small-scale pilots; few such evaluations have been designed as randomised controlled trials. The WSD study was a pragmatic cluster-randomised control trial, representing the largest-scale trial of telehealth and telecare to be carried out in the UK. This presentation focuses on the results of the WSD telecare questionnaire study.

**Objectives:**

To examine the costs and cost-effectiveness of telecare compared to standard support and treatment.

**Methods:**

Economic evaluation conducted in parallel with a pragmatic cluster-randomised controlled trial of Telecare. 2600 people with social care needs participated in a trial of a community-based telecare intervention in three English local authority areas. Approximately half of the participants in the telecare trial also consented to participate in the WSD telecare questionnaire study, which collected information on a number of patient-reported outcome measures and also on the self-reported use of a range of health and social services. Health and social costs were calculated by attaching nationally applicable unit costs to self-reported service use data. The unit costs of telecare support and treatment provided were calculated drawing on administrative and financial data sources and interviews with key informants. The primary outcome for the cost-effectiveness analysis was the quality-adjusted life year (QALY). Secondary outcomes included measures of health-related quality of life and well-being. We employed multivariate analyses to explore the cost-effectiveness of the intervention

**Results:**

The presentation will describe the results of the economic evaluation of telecare, addressing the cost of care and treatment packages used by those participating in the telecare questionnaire study and the results of the cost-effectiveness analysis. These results will be available by the time of the presentation.

**Conclusions:**

These will be available by the time of the presentation.

